# The complete chloroplast genome sequence of the *Dioscorea persimilis* Prain et Burkill (Dioscoreaceae)

**DOI:** 10.1080/23802359.2019.1704645

**Published:** 2020-01-09

**Authors:** Tianxu Cao, Qianglong Zhu, Xin Chen, Putao Wang, Nan Shan, Qionghong Zhou, Yingjin Huang

**Affiliations:** a Ministry of Education of the P.R. China, Key Laboratory of Crop Physiology, Ecology and Genetic Breeding, Jiangxi Agricultural University, Nanchang, China;; b College of Agronomy, Jiangxi Agricultural University, Nanchang, China

**Keywords:** *Dioscorea persimilis*, Chloroplast genome, phylogenetic relationship

## Abstract

*Dioscorea persimilis* belongs to Dioscorea genus, which is considered as one of the most popular food and traditional folk medicine in China. The complete chloroplast genome of *D. persimilis* was determined in this study. The total genome size was 153,219 bp in length, containing a pair of inverted repeats (IRs) of 25,477 bp, which were separated by large single copy (LSC) and small single copy (SSC) of 83,448 bp and 18,817 bp, respectively. The GC content is 37.01%. A total of 129 genes were predicted including 84 protein-coding genes, eight rRNA genes and 37 tRNA genes. Phylogenetic tree analysis of 24 species in the genus Dioscorea indicated that *D. persimilis* was closer to Chinese yam, but has remote phylogenetic relationship with Guinea yam.

*Dioscorea persimilis* Prain et Burkill, belongs to the family Dioscoreaceae and commonly known as ‘Huai-shan-yao’, is one of the important cash crop and traditional folk medicine in southern China. The rhizome of *D. persimilis* contain polysaccharides, saponins and allantoin, which have great pharmacological activities on immunomodulation, antioxidation, blood sugar and blood fat reducing (Yuan 2008). Moreover, *D. persimilis* have been approved as one of the most popular vegetable in China. It is significant for further cultivation, breeding, and utilization to clarify the phylogenetic relationships of species in Dioscorea (Zhou et al. 2018). Their phylogenetic relationship in Dioscorea that contains approximately 600 species has not been better understood (Zhao et al. 2018). The chloroplast genome is smaller in size and has very low recombination, which makes it suitable for species identification and phylogenetic studies (Zhu et al. 2016). Therefore, we characterized the complete chloroplast genome sequence of *D. persimilis* to confirm its phylogenetic position and promote its utilization.

Sample of *D. persimilis* (no. JY236) was collected in Sanming Academy of Agricultural Sciences (26°14′41″N, 117°37′8″E, Fujian, China) and stored in Jiangxi agricultural university (Nanchang, China). The total genomic DNA of *D. persimilis* was isolated from the young leaves using the Plant Genomic DNA Kit (TaKaRa, Beijing, China). The DNA sample was sent to BioMarker (Beijing, China) for constructing a 150 bp pair-end library and being sequenced by illumina HiSeq2500. About 4 Gb of sequence data was obtained after sequencing and base quality control, 0.5 Gb of clean pair-end reads was randomly extracted using Seqtk. The *D. persimilis* chloroplast genome was assembled by using SPAdes (v 3.12.0) (Bankevich et al. 2012), BlastN (v2.7.1), and Gapcloser (v1.12-r6). The paired-end reads were qualitatively assembled with using the Plasmidspades.py in SPAdes. Chloroplast genome sequence contigs were retrieved, ordered and incorporated into a single scaffold sequence by comparison with the chloroplast genome of *Dioscorea alata* (NC_039707.1) using BlastN. The gaps in the chloroplast single draft sequence of were closed by using GapCloser. Chloroplast genome annotation was annotated using CPGAVA2 (Shi et al. 2019) and manually corrected by Sequin and IGV (Robinson et al. 2011).

The complete chloroplast genome of *D. persimilis* (MN585218) is 153,219 bp in length with 37.01% GC contents. The genome was composed of two inverted repeat regions (IRa and IRb) of 25,477 bp, which were divided by a small single copy (SSC) region of 83,448 bp and a large single copy (LSC) region of 18,817 bp. There is a total of 129 genes, including 84 protein-coding genes, eight rRNA genes and 37 tRNA genes. Five of the protein-coding genes, eight of the tRNA genes and four rRNA genes are duplicated within the IRs.

To reveal the phylogenetic position of *D. persimilis*, a phylogenetic analysis was conducted on the complete chloroplast genomes of *D. persimilis* and other 23 species downloaded from the genome database in NCBI. The phylogenetic tree was constructed by Neighbor-Joining method using MAFFT v7.407 (Nakamura et al. 2018) and MEGA v10.0.4 (Kumar et al. 2018). The results demonstrated that *D. persimilis* have closer relationship with *D. alata* and *D. polystachya* that were also known as Chinese yam, but has remote phylogenetic relationship with Guinea yam, *D. rotundata*, that is a staple food in Guinea (Figure 1). These findings further support the conclusions of the previous researches (Wu et al. 2014; Zhao et al. 2018).

**Figure 1.**
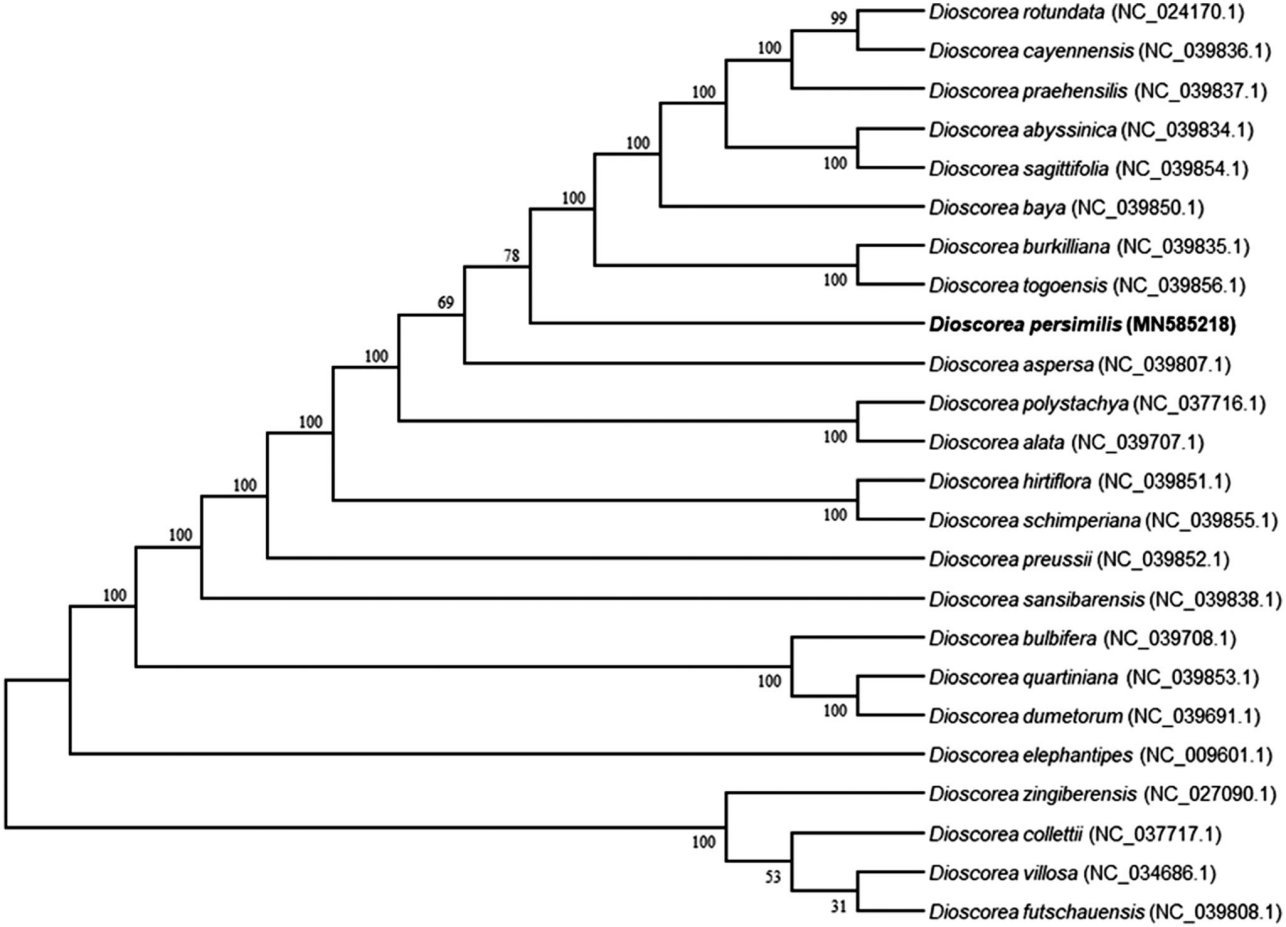
Phylogenetic tree showing relationship of *Dioscorea persimilis* with other 23 species in the genus Dioscorea. Phylogenetic tree was constructed based on the complete chloroplast genomes using Neighbor-Joining method (NJ) with 1,000 bootstrap replicates. Numbers in each the node indicated the bootstrap support values.
